# A Randomized Phase III Trial of Complete Mesocolic Excision Compared with Conventional Surgery for Right Colon Cancer: Interim Analysis of a Nationwide Multicenter Study of the Italian Society of Surgical Oncology Colorectal Cancer Network (CoME-in trial)

**DOI:** 10.1245/s10434-023-14664-0

**Published:** 2023-12-12

**Authors:** Maurizio Degiuli, Aridai H. Resendiz Aguilar, Mario Solej, Danila Azzolina, Giulia Marchiori, Francesco Corcione, Umberto Bracale, Roberto Peltrini, Maria M. Di Nuzzo, Gianandrea Baldazzi, Diletta Cassini, Giuseppe S. Sica, Brunella Pirozzi, Andrea Muratore, Marcello Calabrò, Elio Jovine, Raffaele Lombardi, Gabriele Anania, Matteo Chiozza, Wanda Petz, Paolo Pizzini, Roberto Persiani, Alberto Biondi, Rossella Reddavid

**Affiliations:** 1 Division of Surgical Oncology and Digestive Surgery, Department of Oncology, San Luigi University Hospital, University of Turin, Orbassano (Turin), Italy; 2 Department of Environmental and Preventive Sciences, University of Ferrara, Via Fossato di Mortara, Ferrara, Italy; 3 Department of Surgical Sciences, University of Turin, Turin, Italy; 4 Chirurgia Oncologica e Miniinvasiva Clinica Mediterranea Napoli, University of Naples Federico II, Naples, Italy; 5 Minimally Invasive, General and Oncologic Surgery Unit, University Federico II of Naples, Naples, Italy; 6 ASST Ovest Milanese, P.O. Nuovo Ospedale di Legnano, Legnano, Italy; 7 Minimally Invasive and Gastrointestinal Surgery Unit, Università e Policlinico Tor Vergata, Rome, Italy; 8 Surgical Department, Edoardo Agnelli Hospital, Pinerolo, Italy; 9 IRCCS AOU of Bologna, University of Bologna, Bologna, Italy; 10 AOU of Bologna, Bologna, Italy; 11 Dipartimento Scienze Mediche, Università di Ferrara, Ferrara, Italy; 12 Digestive Surgery, European Institute of Oncology–IRCCS, Milan, Italy; 13 Fondazione Policlinico Universitario A. Gemelli IRCCS, Università Cattolica del Sacro Cuore, Rome, Italy

**Keywords:** Complete mesocolic excision, Right colon cancer, Right hemicolectomy, Embryological plane dissection, Central vascular ligation, Lymphadenectomy, Randomized controlled trial

## Abstract

**Background:**

Although complete mesocolic excision (CME) is supposed to be associated with a higher lymph node (LN) yield, decreased local recurrence, and survival improvement, its implementation currently is debated because the evidence level of these data is rather low and still not supported by randomized controlled trials.

**Method:**

This is a multicenter, randomized, superiority trial (NCT04871399). The 3-year disease-free survival (DFS) was the primary end point of the study. The secondary end points were safety (duration of operation, perioperative complications, hospital length of stay), oncologic outcomes (number of LNs retrieved, 3- and 5-year overall survival, 5-year DFS), and surgery quality (specimen length, area and integrity rate of mesentery, length of ileocolic and middle-colic vessels). The trial design required the LN yield to be higher in the CME group at interim analysis.

**Results:**

Interim data analysis is presented in this report. The study enrolled 258 patients in nine referral centers. The number of LNs retrieved was significantly higher after CME (25 vs. 20; *p* = 0.012). No differences were observed with respect to intra- or post-operative complications, postoperative mortality, or duration of surgery. The hospital stay was even shorter after CME (*p* = 0.039). Quality of surgery indicators were higher in the CME arm of the study. Survival data still were not available.

**Conclusions:**

Interim data show that CME for right colon cancer in referral centers is safe and feasible and does not increase perioperative complications. The study documented with evidence that quality of surgery and LN yield are higher after CME, and this is essential for continuation of patient recruitment and implementation of an optimal comparison.

*Trial registration* The trial was registered at ClinicalTrials.gov with the code NCT04871399 and with the acronym CoME-In trial.

**Supplementary Information:**

The online version contains supplementary material available at 10.1245/s10434-023-14664-0.

Colorectal cancer (CC) is the third most common tumor worldwide and ranks second in terms of death-related cancer/year. Actually, the standard of care is surgery, with 5-year survival rates of 80% or higher for all stages together, excluding stage IV disease.^1^ Nevertheless, the optimal extension of lymphadenectomy remains under debate for both right and left locations. All Western guidelines for the treatment of CC recommend a D2 lymphadenectomy, whereas the Japanese Society for Cancer of the Colon and Rectum guidelines suggest a D3 lymph node (LN) dissection.^2–5^

In 1907 Halsted^6^ stated that LN metastases follow a well-defined and predictable model. According with his theory, the colonic LN metastases first spread to paracolic LNs, then to central LNs, and finally to other organs,^7^ In contrast to the Halsted theory, Fisher^8^ affirmed that LN metastases spread randomly in an unpredictable way. Furthermore, the lymph node ratio plays an important role, as several studies reported that the prognosis of both stages II and III CC depend on the total number of LNs retrieved.

Several authors have reported main differences between right- and left-sided CC in terms of molecular alterations, treatment responsiveness, and survival rates.^9,10^ Consistent with literature data, advanced right-sided (RS) CC has a poorer prognosis than left-sided (LS) CC. This difference could be due not only to their molecular alterations but also to their peculiar lymphatic spread.^9,11,12^ Recently, Kataoka et al.^13^ demonstrated that RSCCs have higher rates of skipped node metastasis than LSCCs. These findings confirmed the previous reports of Nagasaki et al.^14^ showing that central nodes are mostly involved in RSCC compared with LSCC.

In 2009, Hohenberger et al.^15^ introduced the new concept of complete mesocolic excision (CME), which assembles three fundamental items to the standard concept of right colectomy as follows: central vascular ligation (CVL), dissection of the embryologic plane, and resection of a sufficient length of bowel. This procedure aimed to increase the number of LNs retrieved with the routine dissection of central nodes, preserving the integrity of the anterior and posterior sheets of the removed mesocolon.

This new technique suddenly raised a great interest among surgeons, who attempted to demonstrate its superiority over the standard right colectomy. Nevertheless, to date there aren’t enough data supporting its routine adoption for RSCC treatment, in terms of both the number of LNs yielded and the survival rates.^16^ Although a recent systematic review analyzing 21 non-randomized studies reported a total mean number of 27.45 nodes retrieved as well as 5-year overall survival (OS) and disease-free survival (DFS) rates of 84.3% and 82.8% respectively after CME, the authors concluded that the available data did not support the superiority of CME over standard right colectomy due to the limited quality of the evidence. Furthermore, Reddavid et al.^16^ highlighted the lack of a concrete check of surgery quality to assess the compliance of the procedures performed with the CME main issues (number of nodes retrieved, integrity and area of resected mesocolon, length of ileocolic IC and middle colic MC vessels, and eventually, Benz classification).

Very recently two randomized controlled trials (RCT) comparing CME with standard right colectomy have been published. The RELARC study is a multicenter, high-volume, phase 3 superiority trial from China, whereas the Agrusa study is a single-center low-volume trial from Italy.^17,18^ After the analysis of early outcomes, both trials concluded that CME is a feasible and safe procedure that can provide a significantly higher number of nodes retrieved. However, the RELARC trial documented a significantly increased risk of intraoperative major vascular injuries in the CME arm (3% vs. 1%; *p* = 0.045). Neither study has reported any survival results for to date.

Our multicenter trial with a large Western population aims to investigate the three main outcomes of a controlled CME: the quality of surgery (number of nodes retrieved, integrity and area of resected mesocolon, length of IC and MC vessels, and Benz classification), its safety (intra- and postoperative complications), and its efficacy (early oncologic items and survival rates).^19^ In this report, we describe the interim analysis of the study concerning early results (safety and quality of surgery, mostly the number of LNs retrieved). Documentation of a significant increase in the number of LNs removed in the CME arm after enrollment of about 50% of the expected patients also is strongly required by the design of the trial as the essential issue necessary for continuation of patient recruitment.

## Methods

### Study Design

The current study is a randomized, superiority, two-arm, interventional trial involving nine Italian referral centers from the Italian Society of Surgical Oncology (SICO). All consecutive patients with RSCC located between the cecum and proximal third of the transverse colon without distant metastasis were eligible for enrollment in the trial. Patients needed to meet the following inclusion criteria: age 18–85 years, tumors clinically staged as cT2-4aN0 or cT1-4aN+ according to preoperative staging, American Society of Anesthesiologists (ASA) physical status score lower than 4, body mass index (BMI) of 30 kg/m^2^ or lower, and ability to give informed consent. The study excluded patients withdrawing their consent who had distant metastasis diagnosed during surgery or who needed unplanned multiorgan resection. All the inclusion and exclusion criteria are detailed in the previously published protocol.^19^

This study was conducted in accordance with CONSORT guidelines^20^ (CONSORT Checklist-SDC1) (Table S1). The study was ratified by the Institutional Review Board (IRB) of the San Luigi Gonzaga University Hospital and approved later by each IRB of the participating centers. The trial was registered at ClinicalTrials.gov with the code NCT04871399 and with the acronym CoME-in trial.

### Randomization and Blinding

Patients were enrolled in the trial by each participating surgeon after signing the informed consent agreement. The study was a single-blind trial, with only the patients blinded to the surgical procedure.

The coordinating center was responsible for patient allocation (CME or non-CME). The randomization list was managed through a central computerized module. A permuted block randomization of size 30 stratified by the center was centrally implemented. The random assignment sequence was concealed until the procedure was allocated to the patients.

No blinding was applied after group assignment. The assigned arm of the study was communicated to the surgeon only a few minutes before surgery.

### Interventions and Quality Control

The types of surgical interventions are detailed in the previously published study protocol.^19^ The anastomoses were performed manually or mechanically based on each surgeon’s preference. A strict quality control was applied to surgery, pathology, and follow-up evaluation. The participating surgeons were first evaluated by an independent committee of experts who analyzed non-edited video of both the non-CME and CME procedures.

Furthermore, every surgical specimen was carefully measured according to promotor center indications to assess its quality according with Benz classification.^21^ A picture of the specimen was required for documentation and certification of this quality. Following this classification, the surgical specimens were classified as type 0 (true CME) when the dissection was complete, the pedicles of the IC and MC vessels were connected by tissue of the surgical trunk (lymphatic tissue package covering the SMV), and the mesocolic window had a complete medial frame of mesocolic tissue; type 1 when the frame of the mesocolic window was not complete on its medial aspect; type 2 when the frame of the window had a medial and cranial defect; and type 3 when the mesocolic window was not detectable.

Two types of protocol deviation were recognized: “contamination” for non-CME and “noncompliance” for CME. Contamination was the deviation with specimen picture proof of non-CME quality according to Benz classification (Benz <1), and noncompliance was the absence of complete dissection in the CME group according to Benz classification (Benz >0).^21^ Pathologic analysis was conducted in accordance with TNM 8th edition.^22^

The reasons for patient dropout were identification of distant metastases during surgery, a final histopathologic characterization not consistent with the inclusion criteria, and types of surgery performed differently from right hemicolectomy. The follow-up care schedule was reported in the study protocol.^19^

### Outcomes

The primary outcome was 3-year DFS. The primary end point results are not available to date due to the recent starting date of the study.

The secondary end points were as follows:

*Safety:* Duration of operation, intraoperative blood loss, intraoperative complications, postoperative complications, hospital length of stay, and postoperative mortality.

*Oncologic outcomes:* Number of LNs retrieved*,* number of positive LNs harvested, OS (3- and 5-year time frames), and 5-year DFS.

*Quality of CME:* Area of resected mesentery (cm^2^), rate of integrity of both the anterior and posterior mesentery covering layers (%), length from the tumor epicenter to the IC artery (ICA) ligation (cm), length from the tumor epicenter to the right branch of the MC artery (MCA) (or origin of the MCA when requested) ligation, and specimen quality control according to Benz classification (types 0–3).

*Quality of life* (QoL): EORTCQLQ-CR29/QLQ-CR30 and SF36.

### Statistical Analysis

The descriptive statistics are reported by the intervention arm summarizing the continuous data as a median with the interquartile range (IQR). Categorical data are reported as absolute frequencies and percentages. Wilcoxon-type tests were performed for continuous variables and the Pearson chi-square test or Fisher’s exact test, whatever was appropriate, for the categorical variables.

### Interim Analysis

The interim analysis for the interim futility assessment was reported 12 months after the beginning of the study. Based on the results of this interim assessment, the study was stopped if no significant interim increase in LN yield was documented in the experimental arm.^19^ The *p* values for multiple comparisons have been adjusted via Bonferroni^23^ correction.

### Secondary Outcomes

Univariable logistic regression model estimates for the binary end points and linear regression model estimates for continuous ones were performed by considering the intervention as a covariate. Gamma regression models were considered for positive-skew end points. The model results are reported as odds ratios (ORs) with *p* values and 95% confidence intervals (CIs) for the binary outcome. The estimated average mean effect (AME) coefficient is reported with a 95% CI for continuous end points. Analyses were performed using R 3.4.2^24^ with rms^25^ packages.

### Sample Size Calculation

Details of sample size calculation are available in the study protocol.^19^

### Role of the Funding Source

The funding source for this study had no role in study design, data collection, data analysis, data interpretation, or writing of the report.

## Results

**Fig. 1:**
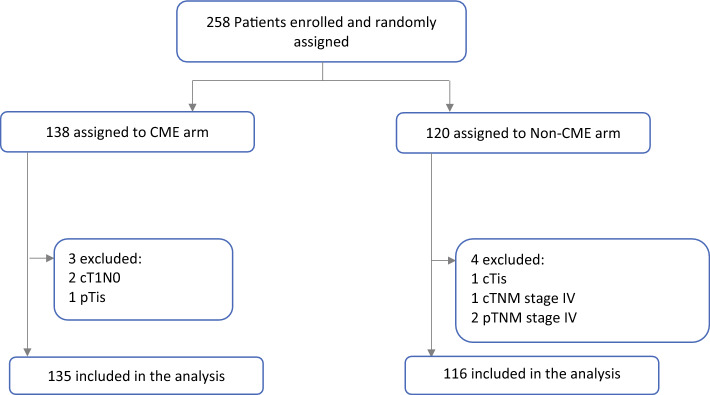
CoME-in trial flowchart.

Between February 2020 and June 2022, the study enrolled 258 patients and randomly allocated them into two groups (120 in the non-CME group and 138 in the CME group) in nine participating centers from the SICO. Seven patients were excluded from the analysis after randomization because they did not meet all the inclusion criteria at the final pathologic examination. Consequently, 251 patients were included in the definitive interim analysis: 116 in the non-CME arm and 135 in CME arm of the study (Fig. 1).

The two groups were well balanced without significant differences in sex, age, BMI, age-adjusted Charlson Comorbidity Index (ACCI), tumor location, and clinical tumor-node-metastasis stage (cTNM) (Table 1).

**Table 1:** Baseline characteristics

	Overall (*n* = 251) *n* (%)	Non-CME (*n* = 116) *n* (%)	CME (*n* = 135) *n* (%)	*p* Value^a^
*Sex*				0.8
Female	125 (50)	59 (51)	66 (49)	
Male	126 (50)	57 (49)	69 (51)	
Median age: years (IQR)	74 (14)	74 (13)	73 (15)	0.6
Median BMI: kg/m^2^ (IQR)	26.0 (4.6)	26.3 (4.5)	25.9 (4.5)	0.5
*ACCI*				0.3
1	1 (0.4)	1 (0.9)	0 (0)	
2	15 (6.0)	8 (6.9)	7 (5.2)	
3	26 (10)	11 (9.5)	15 (11)	
4–5	98 (39)	39 (34)	59 (44)	
6+	111 (44)	57 (49)	54 (40)	
*ASA*				0.4
I	8 (3.2)	3 (2.6)	5 (3.8)	
II	133 (54)	57 (50)	76 (57)	
III	107 (43)	55 (48)	52 (39)	
*Tumor site*				0.8
Ascending	81 (32)	38 (33)	43 (32)	
Cecum	100 (40)	48 (41)	52 (39)	
Distal ascending	1 (0.4)	1 (0.9)	0 (0)	
Hepatic flexure	58 (23)	24 (21)	34 (25)	
Transverse colon proximal third	11 (4.4)	5 (4.3)	6 (4.4)	
*cT stage*				>0.9
cT1	0 (0)	0 (0)	0 (0)	
cT2	70 (30)	33 (30)	37 (30)	
cT3	144 (62)	67 (61)	77 (62)	
cT4	20 (8.5)	10 (9.1)	10 (8.1)	
*cN stage*				0.4
cN+	99 (42)	50 (45)	49 (40)	
cN0	135 (58)	60 (55)	75 (60)	
*cTNM stage*				0.7
I	54 (23)	23 (21)	31 (25)	
II	88 (38)	42 (38)	46 (37)	
III	92 (39)	45 (41)	47 (38)	

The absolute number of cases and percentages are reported for categorical variables and median with interquartile ranges for quantitative ones for the overall, CME, and non-CME groups.

*CME*, complete mesocolic excision; *IQR*, interquartile range; *BMI*, body mass index; *ACCI*, age-adjusted Charlson Comorbidity Index; *ASA*, American Society of Anesthesiologists class; *cT*, clinical tumor stage according to the 8th TNM system; *cN*, clinical ndal stage according to the 8th TNM system; *cTNM*, clinical TNM stage according to the 8th TNM system

^a^Pearson’s chi-squared test; Wilcoxon rank sum test; Fisher’s exact test

The number of LNs retrieved was significantly higher in the CME group than in the non-CME group (25 vs. 20; *p* = 0,012), with an estimated AME of 3.92 (range, 0.85–6.99; Table 2). According to the design of the trial, this finding was essential to continuation of patient recruitment. The number of positive nodes was similar in the two arms.

**Table 2:** Interim analysis assessment

	Overall (*n* = 251)	No-CME (*n* = 116)	CME (*n* = 135)	AME AME (95% CI)	*p* Value
Median no. of lymph nodes retrieved (IQR)	22 (12)	20 (10)	25 (14)	3.92 (0.85–6.99)	0.012

The median number of lymph nodes retrieved with interquartile ranges for the overall, CME, and non-CME groups have been reported. The Gamma model estimated treatment effect (AME) has been reported. The 0.025 Bonferroni adjusted alpha value has been considered to assess a significant difference among groups.

*CME*, complete mesocolic excision; *AME*, average marginal effect; *CI*, confidence interval; *IQR*, interquartile range

Pathologic outcomes are reported in Table 3. The rate of radical resection was comparable between the CME and non-CME groups, with high rates of microscopically margin-negative resection and no gross or microscopic tumor residuals (R0, 99% vs. 95%). The median lengths of the specimen were similar (29 vs. 28 cm). Half of the patients had pathologic stage II disease, whereas 30% had stage III tumor and 19% had stage I tumor, without significant differences between the two arms of the study. Most of the specimens had a moderately differentiated World Health Organization (WHO) grading.

**Table 3:** Pathologic outcomes

	Overall (*n* = 251) *n* (%)	No-CME (*n* = 116) *n* (%)	CME (*n* = 135) *n* (%)	*p* value^a^
*Radicality of resection*				0.051
0	243 (97)	109 (95)	134 (99)	
1	7 (2.8)	6 (5.2)	1 (0.7)	
Median length of the specimen: cm (IQR)	28 (11)	28 (10)	29 (12)	>0.9
*pTNM stage*				0.7
0	0 (0)	0 (0)	0 (0)	
I	43 (19)	23 (21)	20 (17)	
II	118 (51)	54 (49)	64 (53)	
III	70 (30)	33 (30)	37 (31)	
IV	0 (0)	0 (0)	0 (0)	
*pT stage*				0.3
pT1	5 (2.0)	3 (2.6)	2 (1.5)	
pT2	55 (23)	28 (25)	27 (21)	
pT3	157 (64)	67 (59)	90 (69)	
pT4	27 (11)	16 (14)	11 (8.5)	
*pN stage*				0.6
N0	170 (69)	77 (68)	93 (71)	
pN+	75 (31)	37 (32)	38 (29)	
*WHO grading*				0.13
Moderately differentiated	113 (47)	48 (42)	65 (51)	
Poorly differentiated	88 (37)	49 (43)	39 (31)	
Well-differentiated	39 (16)	16 (14)	23 (18)	
No. positive lymph nodes	0.00 (1.00)	0.00 (1.00)	0.00 (1.00)	0.4

The absolute number of cases and percentages have been reported for categorical variables and median with interquartile ranges for quantitative ones for the overall, CME, and non-CME groups.

*CME*, complete mesocolic excision; *IQR*, interquartile range; *pTNM*, pathologic TNM stage according to the 8th TNM system; *pT*, pathologic tumor stage according to the 8th TNM system; *pN*, pathologic nodal stage according to the 8th TNM system; *WHO*, World Health Organization

^a^Fisher’s exact test, Wilcoxon rank-sum test, Pearson’s chi-square test

Evaluation of the fresh specimen quality is detailed in Table 4. The area of resected mesentery was significantly greater in the CME group (106 cm^2^) than in the non-CME group (98 cm^2^). Anterior and posterior mesentery sheet integrity was found in 100% of the CME and 92% of the non-CME specimens (*p* = 0.016). In the CME group, the median lengths of the IC vessels (13 vs. 12 cm; *p* < 0.001) and MC vessels (14 vs. 12 cm; *p* = 0.017) were superior. Most of the CME specimens had a Benz score of 0 (76%), and none had a Benz score of 3, whereas half of the non-CME specimens had a Benz score of 1. Contamination occurred in 24 (20.7%) of 116 patients submitted to the non-CME procedure, and non-compliance occurred in 29 (21.5%) of the 135 patients with CME dissection.

**Table 4:** Fresh specimen evaluation

	Overall (*n* = 251)	Non-CME (*n* = 116)	CME (*n* = 135)	*p* Value^a^
Median area of mesentery: cm (IQR)	100 (64)	98 (48)	106 (73)	0.002
The median integrity of anterior and posterior sheet:% (IQR)	100 (25)	92 (35)	100 (20)	0.016
Median length of IC vessels: cm (IQR)	12.4 (3.0)	12.0 (3.0)	13.0 (4.0)	< 0.001
Median length of MC vessels: cm (IQR)	13.0 (5.0)	12.0 (4.2)	14.0 (4.0)	0.017
Benz score: *n* (%)				< 0.001
0	117 (51)	24 (22)	93 (76)	
I	77 (33)	55 (50)	22 (18)	
II	34 (15)	27 (25)	7 (5.7)	
III	3 (1.3)	3 (2.8)	0 (0)	

The absolute number of cases and percentages have been reported for categorical variables and median with interquartile ranges for quantitative ones for the overall, CME, and non-CME groups.

*CME*, complete mesocolic excision; *IQR*, interquartile range; *IC*, ileocolic; *MC*, middle colic

^a^Wilcoxon rank-sum test, Fisher’s exact test

Finally, an in-depth analysis determined the correlation between the magnitude of protocol deviations and eight patient-, tumor-, and treatment-related characteristics (Table S2). Significant correlations were shown between the type of surgical approach and the incidence of non-compliance in the CME group (*p* = 0.041), with noncompliance significantly higher for open procedures than for minimally invasive dissections. Most of the patients underwent surgery with a laparoscopic approach (85%), whereas 7.6% were treated with a robotic approach and 7.2% had open surgery, with no significant differences observed between the arms (*p* = 0.057). The rate of conversion from minimally invasive to open surgery was very low (5.3%).

Most ileocolic anastomoses were performed with a mechanical intracorporeal technique. The mean duration of surgery was similar in the two groups (CME [176 min] vs. non-CME [172 min]; *p* = 0.6).

In the two arms, the mean blood loss (85 vs. 100 ml; *p* = 0.7) and incidence of intraoperative complications (3% vs. 3%; *p* < 0.9) also were comparable (Table 5). Only one major vascular injury occurred in the CME group, at the site of the superior mesenteric artery (0.7%). The hospital stay was longer in the non-CME group than in the CME group (*p* = 0.039; Table 6).

**Table 5:** Surgical outcomes

	Overall (*n* = 251) *n* (%)	Non-CME (*n* = 116) *n* (%)	CME (*n* = 135) *n* (%)	*p* value^a^
*Type of approach*				0.057
Laparoscopic	214 (85)	96 (83)	118 (87)	
Open	18 (7.2)	13 (11)	5 (3.7)	
Robotic	19 (7.6)	7 (6.0)	12 (8.9)	
Conversion	13 (5.3)	6 (5.4)	7 (5.3)	>0.9
*Anastomotic technique*				0.3
Manual	17 (6.8)	6 (5.2)	11 (8.1)	
Mechanical	234 (93)	110 (95)	124 (92)	
*Anastomosis approach*				0.055
Extracorporeal	38 (15)	23 (20)	15 (11)	
Intracorporeal	213 (85)	93 (80)	120 (89)	
Mean duration of operation (min)	174 ± 76	172 ± 72	176 ± 80	0.6
Mean blood loss (ml)	50 ± 85	50 ± 100	50 ± 85	0.7
Intraoperative complication	8 (3)	4 (3)	4 (3)	>0.9

The Absolute number of cases and percentages have been reported for categorical variables and median with interquartile ranges for quantitative ones for the overall, CME, and non-CME groups.

CME, complete mesocolic excision

^a^Pearson’s chi-square test, Wilcoxon rank-sum test; Fisher’s exact test

**Table 6:** Early postoperative outcomes

	Overall (*n* = 251) *n* (%)	No-CME (*n* = 116) *n* (%)	CME (*n* = 135) *n* (%)	*p* value^a^
Postoperative stay ≥5 days	165 (66)	84 (72)	81 (60)	0.039
Clavien-Dindo				0.5
1–2	40 (56)	19 (53)	21 (60)	
≥3	31 (44)	17 (47)	14 (40)	
30-Day mortality	2 (0.8)	1 (0.9)	1 (0.7)	>0.9
90-Day mortality	4 (1.6)	3 (2.6)	1 (0.7)	0.3
Any early complications (Clavien-Dindo ≥1)	71 (28)	36 (31)	35 (26)	0.4

The absolute number of cases and percentages have been reported for categorical variables and median with interquartile ranges for quantitative ones for the overall, CME, and non-CME groups.

CME, complete mesocolic excision

^a^Wilcoxon rank-sum test, Fisher’s exact test, Pearson’s chi-square test

Postoperative complications (at least one postoperative event) occurred in 35 (26%) of the CME patients and 36 (31%) of the non-CME patients (*p* = 0.4). Moderate and severe complications (Clavien-Dindo ≥3) were reported in 14 (10.4%) CME patients and 17 (14.6%) non-CME patients (*p* = 0.5). Two deaths occurred in the first month after surgery (one in each study arm; *p* > 0.9).

## Discussion

As a new technique, CME remains under investigation. Although renewed attention to meticulous surgical technique certainly has its merits, routine implementation of CME seems currently unfounded for several reasons. First, in contrast to rectal cancer, local recurrence originating from an incomplete or non-optimal removal of colonic mesentery is rare in CC and usually is a manifestation of a systemic disease. Second, although CME may increase nodal counts and therefore staging accuracy, this is unlikely to affect survival because the observed relationship between nodal counts and outcome in CC is most probably not causal but confounded by a range of different clinical variables. Third, several lines of evidence suggest that metastasis to locoregional nodes occurs early and is a stochastic rather than a stepwise phenomenon in CC, reflecting the tumor host-metastasis relationship.^26^ Finally, routine implementation of CME may cause patient harm by longer operating times, major vascular damages, and autonomic nerve injuries.

Available data document that CME with central vascular ligation (CVL) often is a more demanding procedure than standard right hemicolectomy, almost always has a longer duration (10–90 min or longer depending on the completeness of the learning curve), can carry a higher morbidity (longer time to first flatus and major vascular injuries above all), and also is more challenging based on the high variability of vascular anatomy. These issues support the actual debate on the reasons to implement the CME for the treatment of RSCC at least in referral centers.

Above all, three main items currently remain under investigation: the efficacy of CME in increasing the LN yield, the risk of intra- and postoperative complications particularly concerning major vessels injuries, and mostly the relationship between CME and the improvement of survival outcomes.^27^ Another debated point is the identification of measurable indicators to assess the quality of surgery provided, which seems essential to standardization of the procedure. For this purpose, Benz et al.^21^ have recently suggested four main indicators: the length of the specimen, the length of resected IC and MC vessels, the area of the resected mesentery, and its integrity rate.

During the past decade, several systematic reviews and meta-analysis have effectively evaluated these items with the aim to crown CME as the standard of care for RSCC or definitively to reject it.^16,28^ The postoperative outcomes of the studies available to date show acceptable findings. Surgical complications, 30-day mortality, anastomotic leak rate, and reoperation rate are consistent with those observed after standard non-CME right hemicolectomy. Moreover, survival outcomes often are better than those of standard procedures.

Unfortunately, these studies had many limitations. First, most of the studies were case series of a prospective or retrospective nature, and no level 1 evidence from RCTs is available. Second, these studies were not homogeneous concerning the outcomes of interest (safety, quality, oncologic outcomes) because most of them had important missing outcomes. Third, most of the included patients had early-stage disease (60.6% stage 0, I, or II). These limitations represent confounding factors for the final analysis. In the end, most of these studies concluded that well-designed RCTs are necessary to strengthen the evidence base and eventually to justify the implementation of routine use of CME.

Actually, only two RCTs comparing CME and non-CME in RSCC are available in the literature.^17,18^ Both the RELARC^17^ and Agrusa^18^ trials reported a significantly higher number of nodes harvested in the CME arm without any increase in postoperative complications. Otherwise, these studies did not evaluate the quality of surgery.

The current study not only is the third RCT conducted in the world, but also is the first Western multicenter nationwide high-volume phase 3 superiority trial comparing CME with non-CME for RSCC. In this trial, the quality of surgery provided in the CME arm was assessed with the previously reported four indicators. The length of the specimen was similar between the two groups. However, the length of resected ICA and MCA, the area of resected mesentery, and its integrity rate were significantly superior in the CME arm. This observation was documented in all the participating centers, confirming the high quality of surgery performed. The quality of CME provided to the enrolled patients also was evaluated with Benz classification.^21^ We were able to document a significant higher number of Benz score 0 specimens in the CME arm than in the non-CME arm (76% vs. 22%). Neither of the two previous RCTs evaluated the quality of surgery with Benz classification.

Despite strict quality control, noncompliance (see definitions in the Methods section) was identified in 21.5 of the CME procedures and contamination in 20.7% of the non-CME procedures. The only significant correlation was between the magnitude of noncompliance per patient and the type of approach (Table S2). Furthermore, contamination probably occurred as a result of incorrect surgical dissection during the non-CME procedure. Because all the participating surgeons had acquired sufficient experience in CME dissection, they may have had difficulty keeping to the non-CME rules.

These protocol violations may potentially have been responsible, in the intention-to-treat analysis, for an underestimation of the difference in number of LNs harvested between the two arms. However, significant underestimation appears unlikely because although the patients undergoing CME had a moderate grade of noncompliance, the number of nodes yielded was significantly higher in this group than in the non-CME group.

Nevertheless, we should accept the continued occurrence of minor deviations considering the complexity of this trial. Consistent with the design of the study, this interim analysis documented that the median number of LNs harvested in the CME group was significantly higher than in the non-CME group (25 vs. 20). Although a significant relationship between the number of nodes removed and patients’ survival has not been documented with sufficient evidence to date, providing a higher number of nodes with a demanding procedure that includes a super-extended LN dissection is essential to demonstrate any oncologic benefits and to continue enrollment of patients.

Postoperative complications were described as comparable in the CME and non-CME arms of both previous RCTs. However, the RELARC trial^17^ reported that vascular injury was three times more common among the CME patients. This is in line with the reports of several non-randomized studies that documented a significantly increased risk of major vascular injuries during CME surgery, the majority of them at the site of the superior mesenteric vein (SMV), mostly due to central dissection requiring a complete exposure of the SMV and of the Henle trunk. The anatomic variations of this district may certainly contribute to improvement in the risk of major vascular injuries.^17,29^ In the current trial, the prevalence of vascular injury and postoperative complications was comparable in the two groups. The 30- and 90-day mortality rates after surgery were very low and comparable in the two groups.

Although both the Agrusa^18^ and RELARC^17^ trials reported a longer duration of surgery in the CME arm, in our study, the mean time of surgery was only 4 min shorter in the non-CME arm, without any significant difference.

To our best knowledge this trial is the first multicenter high-volume RCT from the West comparing the safety and feasibility of CME with standard right hemicolectomy for patients with RSCC. In this interim analysis, only the short-term outcomes are reported, whereas survival outcomes have not matured to date and are expected in January 2027.

The current trial had some limitations. First, it involved only Italian referral centers for colorectal surgery with experienced surgeons and does not represent the reality of primary care facilities in which surgeons have less expertise. Second, only patients with a BMI of 30 kg/m^2^ or lower were included in this study. Both of these limitations may have contributed to the low perioperative complications and mortality rates and to the short hospital stay and can explain the relatively short duration of the procedure in the CME arm without any significant difference with the standard procedure.

In conclusion, data available from this interim analysis document that the quality of surgery measured by the length of the specimen, the area and integrity rate of the resected mesentery, and the length of IC and MC vessels was higher in CME arm, confirming that CME is an extended procedure compared with non-CME. In addition, the interim data show that CME significantly increases the number of LNs harvested without increasing vascular injuries, postoperative complication rates, postoperative mortality, or length of hospital stay. Hence CME is a safe and feasible technique, particularly when performed by experienced surgeons in referral centers.

###  Supplementary Information

Below is the link to the electronic supplementary material.

Supplementary file 1 (PDF 1598 KB)

Supplementary file 2 (DOCX 28 KB)

## Data Availability

The datasets used and/or analyzed during the current study are available from the corresponding author on reasonable request.

## References

[1] Chang GJ, Hu CY, Eng C, Skibber JM, Rodriguez-Bigas MA (2009). Practical application of a calculator for conditional survival in colon cancer. J Clin Oncol..

[2] Hashiguchi Y, Muro K, Saito Y (2020). Japanese Society for Cancer of the Colon and Rectum (JSCCR) guidelines 2019 for the treatment of colorectal cancer. Int J Clin Oncol..

[3] Colon Cancer AIOM Guideline, 2018.

[4] Argilés G, Tabernero J, Labianca R (2020). Localised colon cancer: ESMO Clinical Practice Guidelines for diagnosis, treatment, and follow-up†. Ann Oncol..

[5] Benson AB, Al-Hawary MM, Azad N, et al. NCCN Guidelines Version 1.2022 Colon cancer continue NCCN guidelines panel disclosures, 2022.

[6] Halsted WS (1907). The results of radical operations for the cure of carcinoma of the breast*. Ann Surg..

[7] Klein CA (2009). Parallel progression of primary tumours and metastases. Nat Rev Cancer..

[8] Fisher B (2008). Biological research in the evolution of cancer surgery: a personal perspective. Cancer Res..

[9] Salem ME, Weinberg BA, Xiu J (2017). Comparative molecular analyses of left-sided colon, right-sided colon, and rectal cancers. Oncotarget..

[10] Fugazzola P, Coccolini F, Montori G (2017). Overall and disease-free survival in patients treated with CRS + HIPEC with cisplatin and paclitaxel for gastric cancer with peritoneal carcinomatosis. J Gastrointest Oncol..

[11] Boeckx N, Koukakis R, de Beeck KO (2017). Primary tumor sidedness has an impact on prognosis and treatment outcome in metastatic colorectal cancer: results from two randomized first-line panitumumab studies. Ann Oncol..

[12] Taieb J, Kourie HR, Emile JF (2018). Association of prognostic value of primary tumor location in stage III colon cancer WithRAS andBRAF mutational status. JAMA Oncol..

[13] Kataoka K, Beppu N, Shiozawa M (2020). Colorectal cancer treated by resection and extended lymphadenectomy: patterns of spread in left- and right-sided tumours. Br J Surg..

[14] Nagasaki T, Akiyoshi T, Fujimoto Y (2015). Prognostic impact of distribution of lymph node metastases in stage III colon cancer. World J Surg..

[15] Hohenberger W, Weber K, Matzel K, Papadopoulos T, Merkel S (2009). Standardized surgery for colonic cancer: complete mesocolic excision and central ligation–technical notes and outcome. Colorectal Dis..

[16] Reddavid R, Osella G, Evola F (2020). Complete mesocolic excision for right colon cancer-state of art: a systematic review of the literature. Ann Laparosc Endosc Surg..

[17] Xu L, Su X, He Z (2021). Short-term outcomes of complete mesocolic excision versus D2 dissection in patients undergoing laparoscopic colectomy for right colon cancer (RELARC): a randomised, controlled, phase 3, superiority trial. Lancet Oncol..

[18] Di Buono G, Buscemi S, Cocorullo G (2021). Feasibility and safety of laparoscopic complete mesocolic excision (CME) for right-sided colon cancer: short-term outcomes: a randomized clinical study. Ann Surg..

[19] Degiuli M, Solej M, Resendiz Aguilar HA, Marchiori G, Reddavid R (2022). Complete mesocolic excision in comparison with conventional surgery for right colon cancer: a nationwide multicenter study of the Italian Society of Surgical Oncology colorectal cancer network (CoME-in trial): study protocol for a randomized controlled trial. Jpn J Clin Oncol..

[20] Antes G (2010). The new CONSORT statement. BMJ..

[21] Benz S, Tannapfel A, Tam Y (2019). Proposal of a new classification system for complete mesocolic excison in right-sided colon cancer. Tech Coloproctol..

[22] Brierley JD, Gospodarowicz MK, Wittekind C (2017). TNM classification of malignant tumours–8th edition. Union Int Cancer Control.

[23] Aickin M, Gensler H (1996). Adjusting for multiple testing when reporting research results: the Bonferroni vs Holm methods. Am J Public Health..

[24] Team RC. R: a language and environment for statistical computing. MSOR Connect. 2014;1.

[25] Harrell FE (2015). Regression modeling strategies.

[26] Willaert W, Ceelen W (2015). Extent of surgery in cancer of the colon: is more better?. World J Gastroenterol..

[27] Koh FH, Tan KK (2019). Complete mesocolic excision for colon cancer: Is it worth it?. J Gastrointest Oncol..

[28] Crane J, Hamed M, Borucki JP, El-Hadi A, Shaikh I, Stearns AT (2021). Complete mesocolic excision versus conventional surgery for colon cancer: a systematic review and meta-analysis. Color Dis..

[29] Bertelsen CA, Neuenschwander AU, Jansen JE (2016). Short-term outcomes after complete mesocolic excision compared with “conventional” colonic cancer surgery. Br J Surg..

