# Supplementary material for: A Randomized Phase III Trial of Complete Mesocolic Excision Compared with Conventional Surgery for Right Colon Cancer: Interim Analysis of a Nationwide Multicenter Study of the Italian Society of Surgical Oncology Colorectal Cancer Network (CoME-in trial)
Source: Ann Surg Oncol. 2023 Dec 12;31(3):1671–80. doi: 10.1245/s10434-023-14664-0 (PMC10838239; doi:10.1245/s10434-023-14664-0)
Supplement: Supplementary file 2 — (DOCX 28 KB) [file 10434_2023_14664_MOESM2_ESM.docx]

**Table 2S** Absolute number of cases and percentages have been reported in the table for categorical variables and median with interquartile ranges for quantitative ones for the overall, CME, and no-CME groups. The patients in the No-CME treatment are the contaminations. Other patients, in the CME arm, are defined as non-compliers if they have a **Benz score>0**

|  | **No CME** | | | | **CME** | | | |
| --- | --- | --- | --- | --- | --- | --- | --- | --- |
| **Characteristics** | **No CME, N = 116^1^** | **Contamination, N = 24^1^** | **Non-contamination, N = 92^1^** | **P-Value^2,3^** | **CME, N = 135^1^** | **Compliance, N = 106^1^** | **Non-compliance, N = 29^1^** | **P-Value^2,3^** |
| **Age** | 74 (13) | 78 (14) | 74 (12) | 0.8 | 73 (15) | 73 (16) | 79 (10) | 0.079 |
| **Gender** |  |  |  | 0.6 |  |  |  | 0.6 |
| F | 59 (51%) | 15 (62%) | 44 (48%) |  | 66 (49%) | 49 (46%) | 17 (59%) |  |
| M | 57 (49%) | 9 (38%) | 48 (52%) |  | 69 (51%) | 57 (54%) | 12 (41%) |  |
| **BMI** | 26.3 (4.5) | 25.2 (5.2) | 26.3 (5.1) | 0.6 | 25.9 (4.5) | 25.9 (4.1) | 25.7 (4.4) | 0.6 |
| **Tumor Location** |  |  |  | 0.8 |  |  |  | 0.8 |
| Ascending | 38 (33%) | 5 (21%) | 33 (36%) |  | 43 (32%) | 35 (33%) | 8 (28%) |  |
| Cecum | 48 (41%) | 12 (50%) | 36 (39%) |  | 52 (39%) | 38 (36%) | 14 (48%) |  |
| Distal Ascending | 1 (0.9%) | 0 (0%) | 1 (1.1%) |  | 0 (0%) | 0 (0%) | 0 (0%) |  |
| Hepatic Flexure | 24 (21%) | 6 (25%) | 18 (20%) |  | 34 (25%) | 27 (25%) | 7 (24%) |  |
| Transverse Proximal Third | 5 (4.3%) | 1 (4.2%) | 4 (4.3%) |  | 6 (4.4%) | 6 (5.7%) | 0 (0%) |  |
| **Surgical Approach** |  |  |  | 0.8 |  |  |  | 0.041 |
| Laparoscopic | 96 (83%) | 22 (92%) | 74 (80%) |  | 118 (87%) | 93 (88%) | 25 (86%) |  |
| Open | 13 (11%) | 1 (4.2%) | 12 (13%) |  | 5 (3.7%) | 1 (0.9%) | 4 (14%) |  |
| Robotic | 7 (6.0%) | 1 (4.2%) | 6 (6.5%) |  | 12 (8.9%) | 12 (11%) | 0 (0%) |  |
| **pT** |  |  |  | 0.8 |  |  |  | 0.2 |
| pT1 | 3 (2.6%) | 0 (0%) | 3 (3.3%) |  | 2 (1.5%) | 1 (1.0%) | 1 (3.6%) |  |
| pT2 | 28 (25%) | 7 (30%) | 21 (23%) |  | 27 (21%) | 17 (17%) | 10 (36%) |  |
| pT3 | 67 (59%) | 14 (61%) | 53 (58%) |  | 90 (69%) | 76 (75%) | 14 (50%) |  |
| pT4 | 16 (14%) | 2 (8.7%) | 14 (15%) |  | 11 (8.5%) | 8 (7.8%) | 3 (11%) |  |
| **pN** |  |  |  | 0.8 |  |  |  | 0.8 |
| N0 | 77 (68%) | 15 (65%) | 62 (68%) |  | 93 (71%) | 72 (71%) | 21 (72%) |  |
| pN+ | 37 (32%) | 8 (35%) | 29 (32%) |  | 38 (29%) | 30 (29%) | 8 (28%) |  |
| **P TNM stage** |  |  |  | 0.8 |  |  |  | 0.5 |
| I | 23 (21%) | 5 (22%) | 18 (21%) |  | 20 (17%) | 12 (13%) | 8 (29%) |  |
| II | 54 (49%) | 10 (43%) | 44 (51%) |  | 64 (53%) | 52 (56%) | 12 (43%) |  |
| III | 33 (30%) | 8 (35%) | 25 (29%) |  | 37 (31%) | 29 (31%) | 8 (29%) |  |
| F: Female; M: Male; BMI: Body Mass Index; pT: pathological Tumour stage according to 8^th^ TNM system; pN: pathological Nodal stage according to 8^th^ TNM system, pTNM: pathological TNM stage according to 8^th^ TNM system;  ^1^Median (IQR); n (%) | | | | | | | | |
| ^2^Wilcoxon rank sum test; Pearson's Chi-squared test; Fisher's exact test | | | | | | | | |
| ^3^Benjamini & Hochberg correction for multiple testing | | | | | | | | |
